# Production of freeze‐dried yeast culture for the brewing of traditional sorghum beer, tchapalo

**DOI:** 10.1002/fsn3.256

**Published:** 2015-07-20

**Authors:** Florent K. N'Guessan, Hermann W. Coulibaly, Mireille W. A. Alloue‐Boraud, Marlène Cot, Koffi Marcellin Djè

**Affiliations:** ^1^ Laboratoire de Biotechnologie et Microbiologie des AlimentsUnité de Formation et de Recherche en Sciences et Technologie des Aliments (UFR‐STA)Université Nangui‐Abrogoua02 BP 801Abidjan 02Côte d'Ivoire; ^2^ Laboratoire d'Ingénierie des Systèmes Biologiques et ProcédésInstitut National des Sciences Appliquées (INSA‐Toulouse)135 Avenue de Rangueil31077Toulouse Cedex 4France; ^3^ Centre Wallon de Biologie Industrielle (CWBI) Unité de Bio‐industries, Université de LiègeGembloux Agrobio‐TechPassage des Déportés 25030GemblouxBelgium

**Keywords:** Freeze‐drying, protective agent, sorghum beer, storage, support material, survival, yeast

## Abstract

Freeze‐drying is a well‐known dehydration method widely used to preserve microorganisms. In order to produce freeze‐dried yeast starter culture for the brewing purpose of African sorghum beer, we tested protective agents (sucrose, glucose, glycerol) in combination with support materials (millet, maize, sorghum, and cassava flours) at 1:1 ratio (v/v). The yeast strains *Saccharomyces cerevisiae* F_12–7_ and *Candida tropicalis* C_0–7_ previously isolated from sorghum beer were used in a mixed culture at a ratio of 2:1 (*C. tropicalis*/*S. cerevisiae*). After the freeze‐drying, the residual water contents were between 0.78 –2.27%, 0.55 –4.09%, and 0.40–2.61%, respectively, with sucrose, glucose and glycerol. The dried yeasts viabilities were between 4.0% and 10.6%. Among the protective agents used, sucrose was found to be the best protectant giving cell viabilities of 8.4–10.6%. Considering the support materials, millet flour was the best support after drying. When the freeze‐dried yeast powders were stored at 4°C and room temperature (25–28°C) for up to 3 months, the survival rates were the highest with cassava flour as the support material.

## Introduction

Sorghum beer is a traditional fermented beverage from most of West African countries where sorghum is produced. It is known as tchapalo in Côte d'Ivoire and by various other names in other African countries. The beers are consumed at various festivals and African ceremonies (e.g., marriage, birth, baptism, the handing over of a dowry, etc.) and constitute a source of economic return for women beer producers. The fermentation of these beverages is uncontrolled and the microorganisms involved come from the raw materials, equipment and local environments or from residues of previous fermentation batches. These microorganisms play an active role in the physical, nutritional, and organoleptic modification of starting materials (Aidoo 1994). However, the wide variety of microorganisms present in a spontaneously fermented food produces a product with widely varying qualities. The use of starter cultures was suggested as the appropriate approach to alleviate the problems of variations in organoleptic quality and microbiological stability of traditional sorghum beer (Sanni 1993; Holzapfel 2002). Thus, various investigations for this purpose have been done (Sefa‐deheh et al. 1999; Orji et al. 2003; Maoura et al. 2005; Sawadogo‐Lingani et al. 2008; Glover et al. 2009; N'Guessan et al. 2010). Beers obtained from inoculation tests with these starters showed organoleptic and sensory characteristics comparable to beers produced in the traditional way. However, these starter cultures were introduced during the fermentation in the form of fresh microbial suspensions which are difficult to maintain for extended periods. To overcome this problem, the use of dried starter cultures, active and stable during storage, is the viable and sustainable solution. Such starter preparations furthermore require the use of cheap raw materials in order to be economically feasible.

Freeze‐drying is a well‐known dehydration method widely used to preserve microorganisms. It is also commonly used in food preservation and for a variety of pharmaceutical applications, including protein‐based drugs (Heller et al. 1997; Cleland et al. 2001). By its ability of combining freezing and drying in a unique operation, this process can create final dried products with the highest quality, but the freeze‐drying step is especially critical as it negatively affects both viability and physiological state of the yeasts (Brashears and Gilliland 1995). The formation of ice crystals induces mechanical damage that leads to cellular death during freezing (Tymczyszyn et al. 2005). Microbial survival during this process depends on many factors, including the intrinsic resistance traits of the strains, initial concentration of microorganisms, growth conditions, and the drying medium (Desmons et al. 1998; Carvalho et al. 2002; Morgan et al. 2006). An enhanced stability of microorganisms can be achieved by adding many protective compounds such as disaccharides, polyols, monosaccharides, skim milk, and other organic molecules (Hubalek 2003). According to the work of Berny and Hennebert (1991), by using skim milk as a support material in combination with two compounds among honey, sodium glutamate, trehalose or raffinose, the viability of *Saccharomyces cerevisiae* cells increased from 30% to 96–98%. Abadias et al. (2001) reported a survival rate of 28.9% for *Candida sake* when 10% skim milk was used. Zhao and Zhang (2005) obtained the highest viability (53.6%) after freeze‐drying of *Oenococcus oeni* H‐2 by using 2.5% sodium glutamate. Generally, each single protectant has its own disadvantages which can be made up by other protectants. Therefore, several protectants are mixed together according to a certain formula for a better performance (Wang 2000).

To date the production of dry starter for the brewing purpose of African sorghum beer remains untapped. Previously, two yeast strains, *Candida tropicalis* C_0–7_ and *S. cerevisiae* F_12–7_ isolated from tchapalo were tested in monoculture and co‐culture at four ratios [2:1, 25:4, 1:4, 2:3 (cells/cells)] for their ability to ferment sorghum wort to produce tchapalo. The co‐culture *C. tropicalis *+ *S. cerevisiae* (2:1) has been suggested as the best starter culture to produce tchapalo (N'Guessan et al. 2010). In this study, we aimed to determine the optimal conditions for freeze‐drying this mixed starter. Various protective agents in combination with supports were tested and freeze‐drying kinetics, water content of freeze‐dried starter cultures, the survival rate after freeze‐drying and during storage, and the strains survival dynamics were evaluated.

## Materials and Methods

### Yeast strains

The yeast strains used in this study were *C. tropicalis* C_0–7_ and *S. cerevisiae* F_12–7_. They belonged to the culture collection of the Food Technology Department (University of Nangui‐Abrogoua, Abidjan, Côte d'Ivoire). They were isolated from traditional sorghum beer from the district of Abidjan (Southern Côte d'Ivoire). They were identified by PCR‐RFLP of the ITS region and sequencing of D1/D2 domains of the 26S rRNA gene (N'Guessan et al. 2011). The yeast strains were maintained routinely at −20°C in 20% glycerol.

### Preparation of culture and support materials

Yeast culture from a Sabouraud‐Chloramphenicol plate was harvested with a loop to prepare a dense suspension in 4 mL of sterile distilled water. The suspension was added to 40 mL of sterile final sorghum wort obtained from one randomly selected traditional brewer at Blockoss (district of Abidjan). The mixture was incubated at 35°C for 24 h. The cells were harvested by centrifugation at 120 *g* for 10 min at 4°C. Harvested cells were washed twice in saline solution (0.85% NaCl) and resuspended in the same solution in order to obtain a 20× concentration factor. The initial cell concentration was then calculated by determining the optical density at wavelength of 650 nm (this wavelength gives the best correlation between the optical density of the culture and the total number of cells it contains).

Four flours (maize flour, sorghum flour, cassava flour, and millet flour) were used as supports. They were purchased from local supplier in Abidjan. Two grams (2 g) of each flour were mixed with 50 mL distilled water and heated to 70–80°C for 20–30 min under agitation and then cooled to 30–40°C.

### Freeze‐drying and storage

Both yeast cultures were mixed at a ratio of 2:1 (*C. tropicalis*/*S. cerevisiae*) and suspended in 25 mL sterile solution of 20 g/L protective agent prepared in distilled water. The protective agents used were sucrose, glycerol and glucose (VWR International, Fontenay Sous Bois, France). Cell suspensions in the three protective agents were blended with support at 1:1 ratio (v/v). After homogenization, samples were transferred into sterilized vials, frozen in a freezer (SANYO, Medical freezer, MDF 235, Gunma, Japan) for 24 h at −40°C and subsequently freeze‐dried with a freeze drier (Alpha 1‐2 Christ; Fischer Scientific, Bioblock, France) at −60°C for 32 h under a vacuum of 6.7–13.3 Pa (50–100 mTorr). Immediately after drying, water content and viability of the freeze‐dried biomass were evaluated.

For the storage experiments, dried yeast powders were packaged into sterile aluminum bags and stored at 4°C and room temperature (25–28°C) for up to 3 months. Samples were taken per intervals of 2 weeks for the determination of cell viability and kinetic of yeast strains.

In order to determine the drying curve, samples were weighed after 1, 2, 4, 8, 16 and 32 h of freeze‐drying at −60°C, in a Mettler AE 200 balance (Grefensee, Zurich) and water content was determined.

### Determination of water content and cell viability

Water content was determined using the modified method described by Audigié et al. (1980). A crucible was dried at 103 ± 2°C, cooled in a desiccator and weighed (m_1_). The crucible was reweighed containing 3 g of powder (m_2_). The crucible and sample were dried in the oven at 103 ± 2°C for 18–20 h, cooled in the desiccator before weighing, redried for 1 h, and reweighed until a constant weight was obtained (m_3_). Calculation:

(1) $$\text{Water content}\;\left(\%\right)=\frac{m_{3}-m_{2}}{m_{1}-m_{2}}\times 100$$

To determine the viable counts of freeze‐dried powder, 0.1 g sample was rehydrated with buffered 0.1% peptone water (BIO‐RAD, France) followed by gentle shaking at room temperature for 20 min. The viability was determined using a pour plate method and the cell suspension was then spread onto Sabouraud Chloramphenicol agar plates (BIO‐RAD, France). After cultivated at 30°C for 72 h, viable cells were enumerated before (initial count) and after freeze‐drying. Colonies at the interval of 30–300 colony‐forming units per plate were counted. All experiments were performed in duplicate. The percentage survival after freeze‐drying was calculated as follows:

(2) $$\text{Viability}=\frac{N}{N_{0}}\times 100$$

where *N* and *N*_0_ represent the viable counts (cfu/g) after freeze‐drying and the initial count (cfu/g) before freeze‐drying, respectively.

During storage, the percentage survival of yeast cells was estimated with the same equation (2) but *N*_0_ represents the counts of viable microorganisms immediately after drying, and *N* the counts of viable microorganisms after a given storage period.

### Identification of yeast strains by ITS‐PCR

At a given storage period, 10 colonies were randomly picked from the countable Sabouraud Chloramphenicol agar plate for PCR amplification. Yeast cells from 48‐h‐old colonies growing on Sabouraud Chloramphenicol agar plate were collected using sterile conditions with the sterile tip of toothpick and suspended in the PCR mixture and directly used for PCR analysis. The amplification of the ITS1‐5.8S‐ITS2 region was carried out in 50 *μ*L of reaction mixture containing 25 *μ*L of PCR Master Mix 2× (Promega, Madison, WI), 1 *μ*mol/L each of forward and reverse primers (ITS1 5′‐TCCGTAGGTGAACCTGCGG‐3′, ITS4 5′‐TCCTCCGCTTATTGATATGC‐3′; Proligo France SAS, Paris, France; White et al. 1990), and 15 *μ*L of nuclease‐free water (Promega). The amplification was performed with a total of 35 PCR cycles in a thermal cycler (Longene^®^ A200 Gradient Thermal Cycler, Longene Scientific Instrument, Hangzhou, China). The cycling program was started with an initial cell lysis at 94°C for 5 min followed by 35 cycles of denaturation at 94°C for 2 min, annealing at 60°C for 1 min, and elongation at 72°C for 2 min. The PCR was ended with a final extension at 72°C for 7 min. Amplified samples were kept at −20°C until further use.

The amplified DNA fragment was separated by applying 10 *μ*L of each PCR product with 2 *μ*L of blue‐orange 6× loading dye (Promega) to 1.4% (w/v) agarose (Promega) gel containing 0.4 *μ*g/mL ethidium bromide (Promega). Approximate sizes of amplicons were determined using a standard molecular weight marker (100‐bp DNA ladder; Promega). The gel was run in 1× TBE buffer (89 mmol/L Tris [Promega], 89 mmol/L Boric acid [Promega], 2 mmol/L Na2‐EDTA [Promega]) for 2 h at 90 V, and photographed using gel documentation system (Eagle Eye II Still Video; Stratagene, California, USA). The expected amplicon sizes were 850 pb for *S. cerevisiae* and 550 pb for *C. tropicalis* (N'Guessan et al. 2011).

### Statistical analysis

The mean values (water content, cell viability) and standard deviation were calculated from two independent experiments. The significance of the difference between the mean values was determined using the analysis of variance (ANOVA) with the software STATISTICA, 99 Edition (StatSoft, Austin, Texas, USA). The confidence interval for a difference in the means was set at 95% (*P* < 0.05) for all comparisons.

## Results and Discussion

### Freeze‐drying kinetics

**Figure 1:**
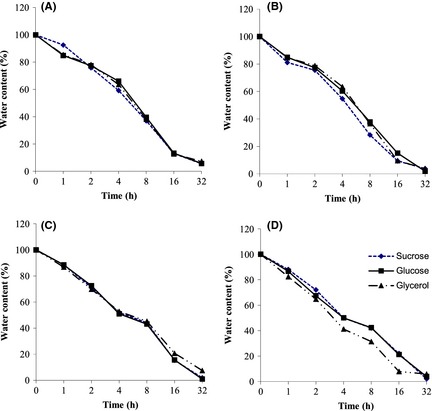
Freeze‐drying curves of the mixed yeast cultures in different protective agents and support materials. (A) millet flour; (B) maize flour; (C) sorghum flour; (D) cassava flour.

Freeze‐drying is known to cause severe damage to organisms at the membrane level as well as to their proteins, but the addition of cryoprotective agents may mitigate injury or inactivation (Hubalek 2003). In this investigation, sucrose, glucose, and glycerol were tested in mixtures with maize, sorghum, millet and cassava flours. According to Nyanga et al. (2012), the preservation of starter cultures by using locally available substrates such as cereals and tubers that are familiar to the consumers, would be compatible with the existing low level of technology in most developing countries. As it can be seen in Figure 1, drying rates were quite similar for all the protective agents when millet flour was used as support material. The water contents after 32 h of drying were 5.6, 5.7, and 7.3%, respectively with sucrose, glucose, and glycerol. Drying rates were also similar for sorghum flour except at the end of the process where drying rate was lower for glycerol. When maize flour was used as a support material, drying was faster with sucrose than with glucose and glycerol. But water contents were under 4% only after 32 h of drying. With cassava flour as a support, medium containing glycerol dried faster than the other protective agents during the first 16 h of drying but the water content remained above 7%. After 32 h of drying, the water contents were lower 4% for sucrose and glucose while it was around 6% for glycerol. For all the tested protective agents, our results also showed that the water contents were between 7.5% and 1% after 32 h of drying. This duration is therefore sufficient for the drying of our starter cultures. In addition, the water contents were generally higher with glycerol than with sucrose and glucose. This observation could be related to the cell‐permeating characteristics of the protective agents used. Glycerol is a permeable compound that penetrates both cell walls and cell membranes while sucrose is an impermeable compound such as lactose, trehalose, mannitol, and sorbitol (Yang et al. 2012). This observation may also indicate that suspending media‐containing sugars may be more porous thereby allowing a faster rate of water mobilization.

### Water content and cell viability of dried yeast cultures

**Figure 2:**
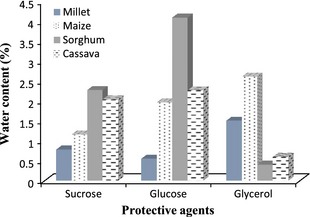
Water content of the freeze‐dried yeast starter cultures in different protective agents and supports materials.

In general for storage, there is an optimum moisture content for each type of organism in a specific protective agent. In this study, three protective agents and four support materials were tested. The beneficial effect of such a support was already suggested by Plessas et al. (2005), who recommended a model of cereal‐based support (starch–gluten–milk matrix) for co‐immobilization of lactic acid bacteria and yeasts for potential use in the food industry. As shown in Figure 2, the residual water contents in the freeze‐dried starter cultures strongly depend on the compounds used. Indeed, with sucrose as protective agent, the residual water contents were between 0.78% and 2.27%. The lowest value was achieved with millet flour while the highest with sorghum flour. When we used glycerol, values were between 0.40% and 2.61%; suspending media containing sorghum and maize gave, respectively the lowest and the highest water content values. The results in this study show lower residual water contents when compared to data of Grabowski et al. (1997). These authors reported a water content of 5–8% for active dry yeasts. Zayed and Roos (2004) reported a value of 2.8% for *Lactobacillus salivarius* while Nagawa et al. (1988) found <3.5% for freeze‐dried cultures of *Bifidobacterium longum*. These observations support the idea that the residual moisture in freeze‐dried materials is directly related to the type of freeze‐drying medium (Greaves 1960).

**Figure 3:**
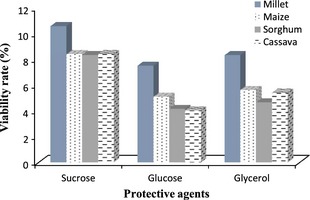
Viability of the freeze‐dried yeast starter cultures in different protective agents and supports materials.

Figure 3 shows the viability of the yeasts after freeze‐drying in sucrose, glucose, and glycerol mixed with millet, sorghum, maize, and cassava flours as support materials. These viabilities were between 4.0% and 10.5%. Our results are in agreement with findings of Abadias et al. (2001). These authors reported *C. sake* viability of 2.8 and 8.6%, respectively for 5 and 10% glucose concentration, 6.2 and 11.4%, respectively for 5 and 10% sucrose concentration. On contrary, the viabilities found in this study were lower than those of several authors (Zayed and Roos 2004; Papavasiliou et al. 2008). The differences observed could be related to the conditions of freeze‐drying. Połomska et al. (2012) demonstrated that one‐step drying at the same temperature of freezing or two‐step drying with main drying at −10°C gave significantly reduced viability results than multistep drying. The yeasts harvest period during culture could also explain these differences. In fact, survival of freeze‐drying is commonly found to be higher for stationary‐phase cells than for exponentially growing cells (Potts 1994; Li et al. 2009). The stationary phase, induced by carbon starvation, triggers a general stress response, which involves induction of a wide range of stress proteins (Hecker and Volker 2001).

Among the protective agents used, sucrose was found to be the best protectant giving cell viabilities of 8.4–10.6% while glucose was the worst protectant (4.0–7.5% of viabilities). This is in line with the results of Carvalho et al. (2004) who found that the sugars that are metabolized were not significantly more effective than those that cannot be metabolized, thus indicating that the effect in stake is of a physicochemical nature. Glycerol, which is a protective agent widely used for freeze‐drying purpose did not provide significant protection during freeze‐drying of yeasts isolated from tchapalo. This result is similar to that obtained by Abadias et al. (2001) for *C. sake*.

Considering the support materials, millet flour was the best support and showed statistically higher survival of the yeasts than maize flour and sorghum flour, respectively. This high survival was probably due to adaptation of cells, or perhaps due to the presence of trace nutrients in the millet flour. Similar observation was made by Papavasiliou et al. (2008) for fresh and fermented whey during the freeze‐drying of kefir culture. So, appropriately assorted support could improve the resistance of tchapalo yeast species to freeze‐drying.

### Cell viability during storage

**Figure 4:**
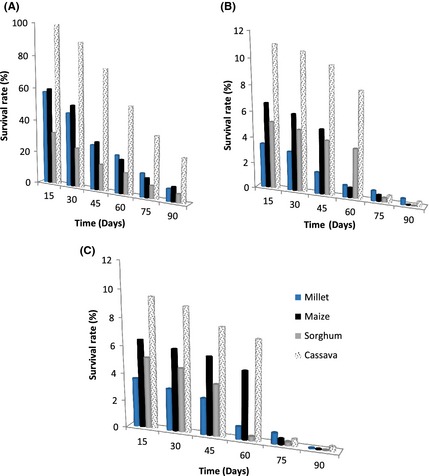
Survival of the freeze‐dried yeast starter cultures in different protective agents and supports materials during the storage at 4°C for 90 days. (A) sucrose; (B) glucose; (C) glycerol.

**Figure 5:**
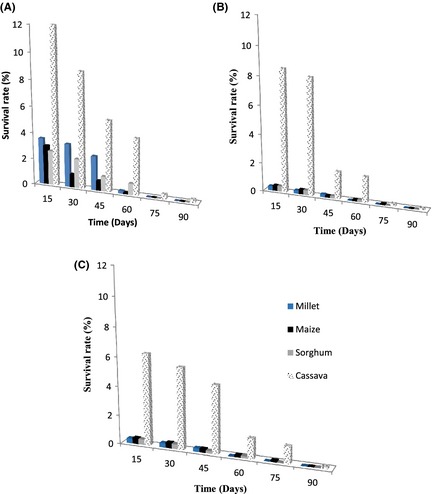
Survival of the freeze‐dried yeast starter cultures in different protective agents and supports materials during the storage at room temperature (25–28°C) for 90 days. (A) sucrose; (B) glucose; (C) glycerol.

The protective agents help to increase cell survival after freeze‐drying and they protect the cultures during storage periods (Desmond et al. 2002). There have been various suggestions related to the loss of viability during storage, and these include cell damage at the cell wall, damage to the cell membrane or damage as a result of membrane lipid oxidation. Different strains of the same species can also differ in their ability to withstand storage. To investigate the effect of storage on survival, freeze‐dried yeast powders were stored at 4°C and room temperature (25–28°C) for up to three months. Figures 4 and 5 report the results. From these figures, it can be observed that regardless of storage temperatures, the survival rates were the highest with cassava flour as support material. Values at 4°C were between 99% and 26.7% for the suspending medium containing sucrose, between 11.0% and 8.1% for glucose, and between 9.5% and 7.2% for glycerol. This could be related to the water activity of each compound used as support material. The results also showed that after 90 days of storage at room temperature, samples showed a higher decrease in viability than those stored at 4°C. Costa et al. (2002) showed that survival rates of *Pantoea agglomérons* CPA‐2 during storage were higher at 4°C than at 25°C. Also, similar results were observed by Champagne et al. (1996) with stored lactic acid bacteria. Low temperatures maintain chemical and biochemical reaction rates at a low level and increase storage stability. This is why 0–5°C is usually the temperature range chosen for storage (Selmer‐Olsen et al. 1999). Furthermore, increased survival of freeze‐dried cultures at low temperatures may be due to a reduction in the rate of unsaturated fatty acid oxidation (Castro et al. 1995). Hence, according to several authors, to prevent/reduce such an oxidative phenomenon, thus providing increased survival during storage, dried powders should be stored under vacuum (Castro et al. 1995) or under controlled water activity (Teixeira et al. 1995) and stored in darkness.

A significant decrease of yeast viability during storage occurred in most of the preparations, especially in those obtained with glycerol, where cells survival decreased even to <0.1% after 90 days of storage. The least decrease of cell viability was observed in sucrose preparations. After 60 days of storage at 4°C, viabilities rates were 53.5% for sucrose + cassava flour preparation while they were 8.1 and 7.2%, respectively, for glucose + cassava flour and glycerol + cassava flour media. Sucrose which was the best protective agent for freeze‐drying is also the best protective agent for the dried yeasts stability during the storage period but it was not the same for millet flour during storage. The results basically confirm the literature (Crowe et al. 1987).

The kinetic of the yeast species used for the starter cultures formulation in this study (S*. cerevisiae* and *C. tropicalis*) showed that these two yeasts were still viable in the freeze‐dried culture for cassava flour as support material during storage (results not shown). On the contrary, with millet flour as the support material, we found *C. tropicalis* only in the dried powder during storage. So, the preparation sucrose + cassava flour was suggested as the appropriate medium for the freeze‐dried mixed culture of tchapalo.

## Conclusion

Our study was focused for the first time on freeze‐drying preservation of yeast strains, potential starter culture for tchapalo production. We tested the use of cheap raw materials in mixture of protective agents in order to be economically feasible for the African people. The results showed that the suspending medium used greatly influenced the survival of freeze‐dried yeasts. From the three protective agents and four support materials analyzed, sucrose and millet flour were, respectively, the best protective agent and support material during the freeze‐drying. The best storage medium was observed with the mixture of sucrose and cassava flour. So for the production of freeze‐dried starter for the brewing purpose of tchapalo, sucrose + cassava flour was suggested as the appropriate medium. This starter culture can be stored at 4°C for at least 60 days and up to 45 days at room temperature. However, before its use by traditional brewers, other investigations are need in order to optimize yeast survival during the freeze‐drying process.

## Conflict of interest

None declared.
